# The Mechanism of Action of Antigen Processing Independent T Cell Epitopes Designed for Immunotherapy of Autoimmune Diseases

**DOI:** 10.3389/fimmu.2021.654201

**Published:** 2021-04-14

**Authors:** Ella R. Shepard, Anja Wegner, Elaine V. Hill, Bronwen R. Burton, Sarah Aerts, Evelien Schurgers, Brecht Hoedemaekers, Sky T. H. Ng, Heather B. Streeter, Lotta Jansson, David C. Wraith

**Affiliations:** ^1^ School of Cellular and Molecular Medicine, University of Bristol, Bristol, United Kingdom; ^2^ Apitope International NV, Diepenbeek, Belgium; ^3^ Institute of Immunology and Immunotherapy, University of Birmingham, Birmingham, United Kingdom

**Keywords:** apitope, immunotherapy, immunological tolerance, interleukin-10, dendritic cell, autoimmune disease, Tr1 cell, synthetic peptide

## Abstract

Immunotherapy with antigen-processing independent T cell epitopes (apitopes) targeting autoreactive CD4^+^ T cells has translated to the clinic and been shown to modulate progression of both Graves’ disease and multiple sclerosis. The model apitope (Ac1-9[4Y]) renders antigen-specific T cells anergic while repeated administration induces both Tr1 and Foxp3^+^ regulatory cells. Here we address why CD4^+^ T cell epitopes should be designed as apitopes to induce tolerance and define the antigen presenting cells that they target *in vivo*. Furthermore, we reveal the impact of treatment with apitopes on CD4^+^ T cell signaling, the generation of IL-10-secreting regulatory cells and the systemic migration of these cells. Taken together these findings reveal how apitopes induce tolerance and thereby mediate antigen-specific immunotherapy of autoimmune diseases.

## Introduction

There are many forms of potentially life-threatening autoimmune diseases that collectively impact up to 10% of individuals at some time in their life. Current approaches to immunotherapy of autoimmune diseases use non-specific immunotherapeutic drugs to treat the disease. These drugs fail to address the underlying cause of immune pathology, loss of tolerance to self-antigen/s, and expose the individual to a higher risk of infectious disease and cancer. Most novel approaches to antigen-specific immunotherapy aim to induce tolerance in autoantigen-specific CD4^+^ T cells since CD4^+^ T cells orchestrate the adaptive immune response (1, 2). Among all of the developing approaches for specific immunotherapy, the most direct and straightforward is tolerance induction with synthetic peptides based on CD4^+^ T cell epitopes (1, 3).

We have shown that some but not all T cell epitopes will induce tolerance to self-antigens. The first rule is that synthetic peptides must mimic the conformation of the naturally processed antigen in order to engage self-antigen reactive T cells and induce tolerance (4). Tolerogenic peptides are, therefore, designed as antigen-processing independent T-cell epitopes (apitopes) that bind to MHC II in the correct conformation and ligate the T cell receptor (TCR) on such cells. The second rule is that peptides must be soluble for effective tolerance induction (5, 6). Apitopes have been designed to modulate disease progression in two autoimmune diseases with distinct immune pathologies, Graves’ disease and multiple sclerosis (7, 8). The successful outcome of early clinical trials in these diseases imply that apitopes based on known self-antigens will be appropriate treatments for other autoimmune conditions where the target antigens are known.

Here we investigate why tolerogenic peptides need to be designed as apitopes and why they depend on solubility. We reveal that a model apitope, Ac1-9[4Y], based on the N-terminal epitope of myelin basic protein, binds directly to steady state dendritic cells (DC) rather than other antigen presenting cells (APC) in lymphoid organs. The outcome of apitope treatment is the induction of antigen-specific Tr1 cells that are both anergic and immunoregulatory through secretion of interleukin-10 (IL-10) (9–12). Tr1 cells were first described by Groux (13) and colleagues, are distinct from Foxp3^+^ regulatory T (Treg) cells (14, 15) and mediate suppression through secretion of IL-10 (16, 17). Here we show that tolerance induction results in a membrane proximal block in cell signaling associated with T-cell anergy and Tr1 cell generation.

Our evidence shows that tolerance induction and the generation of the predominant immunoregulatory Tr1 cell population occurs in secondary lymphoid organs. Here we use an IL-10 reporter mouse derived from the ‘tiger’ reporter (18) to demonstrate that tolerance induction results in migration of the resulting IL-10 secreting regulatory T-cells to peripheral tissues including the liver and CNS. These results address key remaining questions relating to the mechanism of action of apitopes including how they induce both anergy and Tr1 cell differentiation and the systemic impact of the resulting tolerance.

## Materials and Methods

### Mice

B10.PL (JAX mice), Tg4 (19), Tg4.CD45.1^+^, IL-10 reporter tiger-Tg4 (20) and HLA-DR3 (21) mice were bred under SPF conditions at the University of Bristol or Innoser (Diepenbeek, Belgium). Experiments in the UK were conducted under Home Office project license 30/3195 while studies with HLA-DR3 mice studies were approved by the ‘Ethical Committee for Animal experiments’ (ECD) at Hasselt University.

### Peptide Antigens

Properties of the peptides used in this study including Grand average of hydropathy (http://www.gravy-calculator.de/) are shown in **Table 1** and **Supplementary Table 1**. All MBP peptides were synthesized by GL Biochem (Shanghai) Ltd (GLS) while TSHR peptides were prepared by Severn Biotech Ltd (Severn, Kidderminster, Worcs, UK) or Genscript (Leiden, The Netherlands). Peptides were >95% purity.

**Table 1:** Properties and sequences of modified MBP peptides.

	**Peptide name**	**Sequence**	**GRAVY score**
**Native MBP sequence**	Ac1-9[4K]	Acetyl-ASQ**K**RPSQR-amide	-2.367
**High affinity analogue**	Ac1-9[4Y]	Acetyl-ASQ**Y**RPSQR-amide	-2.078
**High affinity analogues increasing in hydrophobicity**	4Y-LF	Acetyl-ASQ**Y**RPS**LF**-amide	-0.456
	4Y-2LF	Acetyl-ASQ**Y**RPS**LFLF**-amide	0.227
	4Y-3LF	Acetyl-ASQ**Y**RPS**LFLFLF** -amide	0.700

In bold: indicate the amino acids changed in the peptides.

### Peptide Solubility

Peptides were stored lyophilized at 4°C and reconstituted in either PBS or dimethyl sulfoxide (DMSO) (Sigma-Aldrich) at 4mg/ml, or equivalent millimolar concentration. Serial dilutions were made in either PBS or DMSO, depending on initial reconstitution. Visual observations were made of turbidity after 16 hours at room temperature. Samples were spun at 16,280 x g for 10 minutes, as specified. Measurements of absorbance at 280nm and 340nm were taken using a Nanodrop 2000 spectrophotometer (Thermo Fisher Scientific), eight readings (or as specified) were taken from each condition, with separate samples used for each reading. Aggregation index was calculated by the formula AI = 100 x (Abs340/(Abs280-Abs340)), giving a measure of aggregation in a solution. Absorbance at 280nm is suitable for peptides containing amino acids with aromatic rings that absorb the wavelength 280nm (phenylalanine (F), tryptophan (W) or tyrosine (Y)).

For dynamic light scattering, peptides were reconstituted in PBS or water and allowed to sit for at least 8 hours at the specified concentration. Samples were spun in a benchtop microfuge at 13000rpm for 10 minutes. 100μl of supernatant was taken into semi–micro cuvettes (BRAND, Sigma-Aldrich) which had been cleaned with pure nitrogen. The samples were run on a Malvern Zetasizer machine (Malvern Instruments Ltd., Malvern, UK).

### Soluble Peptide Challenge and Dose Escalation Tolerance Induction

Tg4 mice were primed with a single dose (80μg) of 4Y in PBS by SC injection. Tolerance induction involved escalating dose immunotherapy (EDI) whereby each Tg4 or tiger-Tg4 mouse was treated with 0.08, 0.8, 8, 80, 80 and 80μg 4Y in PBS by SC injection at the right-side flank every 3^rd^ or 4^th^ day (20).

### Cell Purification and Flow Cytometry

CD11c^+^ cells were isolated from whole splenocytes in MACS buffer using the CD11c microbead positive selection kit (Miltenyi Biotec, Surrey, UK) as per the manufacturer’s instructions. Single cell suspensions were used for magnetic selection using the EasySep Mouse B Cell Isolation kit or EasySep Mouse Monocyte Enrichment kit (both Stemcell Technologies, Grenoble, France) as per the manufacturer’s instructions. In the B10.PL APC targeting experiments, single cell suspensions (without digestion) of Tg4 splenocytes were counted and T cells isolated using Magnisort^®^ Mouse CD4 T cell isolation kit (affymetrix, eBioscience, Hatfield, UK) as per the manufacturer’s instructions. Cells were either enriched by magnetic sorting prior to fluorescence-activated cell sorting (FACS) or sorted from splenocytes. CD11c^+^ were enriched by MACS isolation from B10.PL mice treated with a single 80µg dose of MBPAc1-9[4Y], or 200µl of PBS. Cells were stained for CD4 (GK1.5, eBioscience) and CD8 (53-6.7, Biolegend) expression and sorted into CD11c^+^ subsets using a BD Influx™ flow sorter. Peptide bound to APC was detected *in vitro* by activation of naïve Tg4 cells, as evidenced through ^3^H.thymidine incorporation. In the DR3-transgenic mouse APC targeting experiments, CD11c^+^ cells were enriched by MACS isolation from mice that had received a single SC injection of 80µg of the 5D-K16 peptide (see **Table 1**). Peptide bound to APC was detected *in vitro* by activation of CD4^+^ T cells from the lymph nodes and spleen of HLA-DR3 transgenic mice immunized with the 5D peptide in CFA. T cell activation was detected by interferon gamma secretion over 3 days.

### Cell Transfer Protocol

Cells were isolated as specified and transferred to mice by intraperitoneal (IP) injection in a volume of 200µl. On day -1 splenic CD4^+^ T cells were isolated from untreated Tg4.CD45.1^+^ mice by magnetic separation. Cells were resuspended in PBS at 5x10^6^ per mouse for IP injection to untreated B10.PL mice.

On day 0, CD11c^+^ cells were isolated by magnetic selection from splenocytes 2 hours following a single s.c. dose of 80µg MBP Ac1–9 [4Y] or 200µl PBS. 1x10^6^ of enriched CD11c^+^ cells were transferred per mouse to B10.PL mice that had received CD4^+^ cells from Tg4.CD45.1^+^ spleen (see above). Repeated CD11c^+^ transfers were performed on day +5 and +10 after the first CD11c^+^ transfer. Mice were sacrificed and spleens harvested 3-4 days following the final CD11c^+^ cell transfer, before being processed for surface staining, intracellular cytokine staining (ICCS) and proliferation assessment by tritiated thymidine (^3^H-TdR) incorporation at the time specified.

### Cell Proliferation and Cytokine Measurement

Proliferation was assessed by tritiated thymidine (^3^H-TdR) incorporation with either whole splenocytes or isolated APC populations and antigen-specific Tg4 responder CD4^+^ T cells. Tg4 whole splenocytes were cultured in 96-well round bottom plates, 2x10^5^ whole splenocytes per well, with addition of the indicated concentration of the relevant peptide. Whole splenocytes from B10.PL mice that had received CD4^+^ CD45.1^+^ T cells followed by CD11c^+^ cells were cultured in 48–well plates at 1.25x10^6^ per well. Isolated populations were plated out at 5x10^4^/well CD4^+^ T cells, with either equal numbers of CD11c^+^ DC, B cells or monocytes enriched as specified. Cells were cultured at 37°C, 5% CO_2_. At 24 and 72 hours, supernatant was taken for cytokine analysis and medium was supplemented with 0.5µCi ^3^H-TdR (GE Healthcare Lifesciences, UK) for 16 hours. Plates were frozen at –20°C in preparation for later harvesting with a Tomtec harvester onto filter papers (Cox Scientific, Kettering, UK). Levels of incorporated radioactivity were assessed from the dried filter papers, sealed in sample bags (Wallac, Milton Keynes, UK) with 3ml scintillation fluid, ^3^H-TdR incorporation was measured on a 1450 MicroBeta liquid scintillation counter (Wallac).

MFBI Th1/Th2 Flow Cytomix Multiplex kits (eBioscience) were used to measure the concentration of cytokines in the serum 2 h after s.c. treatment with soluble peptide.

Fluorescence intensity was measured on a FACS Calibur flow cytometer (BD Biosciences), and data were analyzed using FlowCytomix Pro software (eBioscience). Conventional sandwich enzyme-linked immunosorbent assays were performed to quantify cytokine concentration in cell culture supernatant (harvested at 24 h after re-stimulation for IL-2, at 72 h for IFN-γ and IL-10) using matched antibody pairs (all BD Biosciences). IL-2; coating, JES6-1A12 (2µg/ml), biotinylated, JES6-5H4 (0.5µg/ml). IFN-γ; coating, R4-6A2 (2µg/ml), biotinylated, XMG1.2 (0.5µg/ml). IL-10; coating, JES5-2A5 (2µg/ml), biotinylated, SXC-1 (0.5µg/ml). Optical change was measured with a SpectraMax 190 microplate reader (Molecular Devices); cytokine concentration was calculated using Microplate Manager software (Bio-Rad).

Intracellular cytokine staining of splenocytes was performed after a 3-h stimulation with phorbol 12-myristate 13-acetate (5ng/ml) and ionomycin (500ng/ml) (both Sigma-Aldrich) with GolgiStop (BD Biosciences). Cells were stained with Vβ8-FITC (clone KJ16-133, diluted 1:100) or with fixable viability dye eFluor780 (1:1,000) before surface staining with CD4-Alexa700 (GK1.5, 1:100) and fixation using IC fixation buffer (all from eBioscience). Antibodies for intracellular cytokine staining were diluted in permeabilization buffer; IL-10-allophycocyanin (APC) (JES5-16E3, 1:200), IFN-γ-PerCP-Cy5.5 (XMG1.2, 1:200) (both from eBioscience). Data were collected using an LSR II flow cytometer (BD Biosciences) and analyzed using FlowJo software (Treestar).

### Induction of EAE

EAE was induced in Tg4 mice by s.c. injection at the tail base of 100 μl of a sonicated emulsion containing equal volumes of CFA and either 1 mg of spinal cord homogenate suspended in PBS or 200 μg of MBP Ac1-9[4K] in PBS. CFA was supplemented with 4mg/ml heat-killed *Mycobacterium tuberculosis* (both from Difco). Pertussis toxin (200 ng) (Sigma-Aldrich) was administered i.p. in 500 μl of PBS on days 0 and 2. Individual mice were monitored daily for EAE and scored as follows: 0, no disease; 1, flaccid tail; 2, hindlimb weakness and/or impaired righting; 3, hindlimb paralysis; 4, hind and forelimb paralysis; 5, moribund or dead.

### Western Blotting

Proteins were extracted in 50 mM Tris, 120mM NaCl, 1 mM EDTA with 1% IGEPAL CA-630 and protease and phosphatase inhibitor cocktails (Thermo) before SDS-PAGE. Western blotting was conducted using standard techniques. Proteins from cellular lysates were separated on 10% SDS-PAGE NuPAGE gels (Invitrogen, Carlsbad, CA). Following electrotransfer to supported Immobilon-P PVDF Membrane (Merck Life Science UK Ltd. Dorset, UK), blots were blocked with 5% non-fat dry milk in TBS, 0.1% Tween-20 for 1 h at room temperature. Blots were then incubated overnight at 4°C with antibodies against the following proteins: ERK2 or GAPDH, phospho-ERK, phospho-STAT3 and phospho-STAT5 (Cell Signaling Technology, London, UK). The blots were washed with TBS-Tween-20 (0.1%) followed by incubation with anti-rabbit IgG, HRP linked antibody (Cell Signaling Technology, London, UK). The blots were developed using an ECL chemiluminescence detection kit (Cytiva).

### Statistics

Cell proliferation data was transformed using the transformation Y=√(Y) followed by Y=Log (Y). Proliferation, serum cytokine, cytokine ELISA and peptide aggregation data was analyzed by 2-way ANOVA followed by multiple comparisons using the Bonferroni *post hoc* test of significance. For flow cytometry analysis, the Student’s t-test for pairwise comparison or one-way analysis of variance (ANOVA) with Sidak’s *post hoc* test was used for the comparison of more than two groups. Otherwise, the nonparametric equivalents of the t-test the Mann-Whitney U test or the nonparametric equivalent of ANOVA the Kruskal-Wallice with Dunn’s *post hoc* test was used. For sample sizes of three, a nonparametric test was always chosen for comparison.

## Results

### Antigen-Presenting Cells Targeted by Tolerogenic Peptides

**Figure 1:**
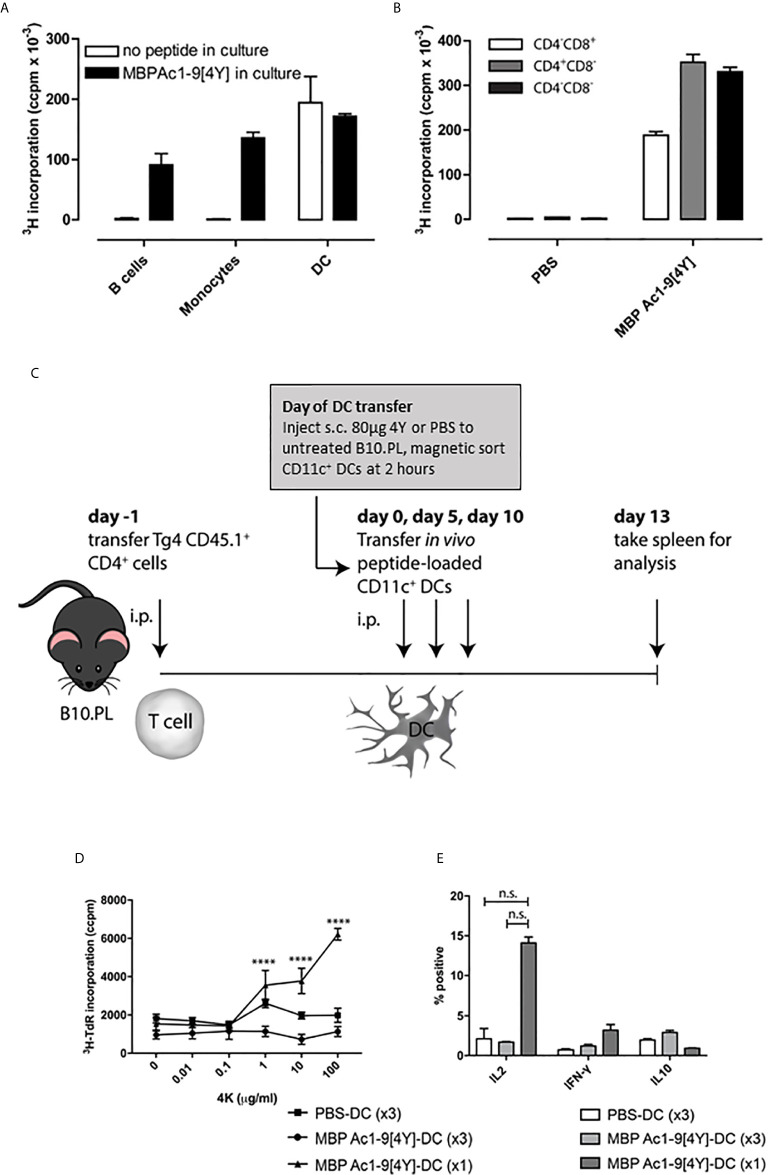
Soluble peptide epitopes target steady-state DC *in vivo*: B10.PL mice received a SC injection of 80µg 4Y or PBS alone (not shown). After 2h, splenic APC populations were enriched by FACS. **(A)** sorted APCs were cultured in a 96-well plate at 5x10^4^ per well with equal numbers of CD4^+^ T cells isolated from an untreated Tg4 mouse. Open bars represent cells from mice that received 80µg MBP Ac1-9[4Y] SC, filled bars show proliferation of cultures with 4Y added *in vitro* (1µg/ml). Proliferation was measured by tritiated thymidine (^3^H-TdR) incorporation at d3. Graphs show mean of triplicate wells and error bars show SEM. **(B)** as **(A)**; however, CD11c^+^ DC from mice treated with 4Y or PBS alone were fractionated by FACS into major subsets before culture with Tg4 CD4^+^ T cells. **(C)** protocol for cell transfer experiments. **(D)** 4Y–DC (x3) and PBS–DC (x3) group received three transfers of 1x10^6^ CD11c^+^ DCs from mice pre-treated with 4Y or PBS respectively on days 0, 5 and 10. 4Y–DC (x1) received MBP Ac1-9[4Y]-loaded DCs on day 10 only. Splenocytes from individual mice were cultured at 2.5x10^6^/ml with Ac1-9[4K] for 72 hours, proliferation was measured by tritiated thymidine (^3^H-TdR) incorporation. Graph shows mean of four repeat wells per mouse (n=2 mice per group), error bars show SEM (*p ≤ 0.05, **p ≤ 0.01, ***p ≤ 0.001, ****p ≤ 0.0001). **(E)** graphs of % of IL-2, IFN-γ and IL-10 expressing cells following ICCS staining protocol. Data represents mean value for two samples per treatment group, error bars = SEM, n.s. = not significant.

We have shown previously that antigen-specific immune responses can be modulated by administration of soluble peptide *via* intranasal (IN) (9) or subcutaneous (SC) administration (20). These peptides must behave as antigen processing independent epitopes (apitopes) to induce effective tolerance (4). Our studies have shown that repeated IN or SC administration of a model apitope, the high affinity analogue of the N-terminal epitope of myelin basic protein Ac1-9[4Y] (abbreviated to 4Y), in the TCR transgenic Tg4 mouse leads to transient T cell activation followed by T cell anergy and the generation of IL-10 secreting Tr1 cells (20, 22). A single administration of 4Y leads to secretion of IL-2 into the serum of naïve mice with levels of cytokine peaking at 2h. This implies that injection of soluble peptide leads to rapid activation of T cells in lymphoid organs. Indeed, we originally showed that peptide administered IN was detectable on splenic and lymph node APC at 20 mins, as evidenced by the ability of the APC to present the peptide to naïve T cells *in vitro*, with levels of APC loading reaching a peak between 1 and 4h (23). Here we investigate which APC in spleen and lymph node are targeted by apitopes. We gave a single SC dose of 4Y known to induce secretion of cytokines into blood and then enriched different APC subsets from the recipient spleen at 2h, the time of peak cytokine secretion. As shown in **Figure 1A**, splenic CD11c^+^ DC were the only major APC clearly capable of binding peptide administered by the SC route whereas neither monocytes nor B cells were targeted by soluble peptide *in vivo*. As a control, we showed that all three APC populations were able to present 4Y when the peptide was added back to co-cultures *in vitro*. After identifying the APC population presenting soluble 4Y peptide *ex vivo*, we next sought to establish whether splenic CD11c^+^ DC subsets loaded peptide equally. Three subsets of CD11c^+^ splenic DCs have been defined by their expression of CD11c, CD11b, CD4 and CD8 (24, 25). CD11c^+^ CD8^-^ CD11b^+^ cells have been shown to be involved in i.v. tolerance (25), suppression of EAE (26) and presentation of exogenous antigens efficiently on MHC class II (27), whereas the CD11c^+^ CD8^+^ population is associated with MHC class I presentation of endogenous antigens (25). Three subsets of CD11c^+^ cells were isolated by FACS from B10.PL mice after SC 4Y peptide administration, or PBS control: CD4^-^CD8^+^, CD4^+^ CD8^-^ and CD4^-^ CD8^-^. The CD11c^+^ populations were assessed for *in vivo* loading of 4Y by their ability to induce proliferation in untreated Tg4 CD4^+^ T cells *in vitro*. The CD11c^+^ subsets all presented peptide; however, both CD8^-^ DC populations induced more proliferation than the CD8^+^ subset, whether CD4^+^ or CD4^-^ (**Figure 1B**). This agrees with previous findings that demonstrated CD8^-^ CD11c^+^ splenic DCs are more efficient at presentation of antigen on MHC II (27).

We next investigated what impact either a single or repeated injection of DC collected from mice treated with soluble 4Y would have on the immune responsiveness of T cells following transfer into naive recipients (**Figure 1C**). B10.PL mice were seeded with CD4^+^ cells from CD45.1^+^ Tg4 mice and rested for 24h before receiving either a single dose of DC from 4Y treated mice or 3 doses of DC from mice treated either with PBS or 4Y. As shown in fig. 1D, mice receiving control DC from mice treated with PBS showed little cell proliferation or cytokine secretion. Mice given a single dose of 4Y-DC were primed and showed enhanced IL-2 production and proliferation. Both priming for IL-2 secretion and cell proliferation were abrogated, however, by repeated administration of 3 doses of 4Y-DC (**Figures 1D, E**). The *in vivo* response to transferred 4Y-DC, therefore, recapitulates the impact of repeated administration of peptide alone whereby a single dose leads to priming of IL-2 secretion which is suppressed with repeated dosing (9).

### Role of Peptide Solubility in Peptide-Induced Tolerance Induction

Previously we have shown that the impact of CD4 T cell epitope administration in the absence of adjuvant depends on peptide solubility. An insoluble peptide epitope (861-874) from the red blood cell band 3 protein was shown to promote autoimmunity when administered intranasally in the NZB model of autoimmune hemolytic anemia (5). Importantly, however, addition of charged residues at the N- and C-termini of the band 3 epitope rendered it both soluble and immuno-modulatory. Unlike the insoluble 861-874 that both primed band 3 reactive inflammatory T cells and promoted generation of pathogenic anti-erythrocyte antibodies, the more soluble derivative (861-875 (Glu861, Lys875)) did not induce inflammatory T cells and prevented development of anemia (5).

**Figure 2:**
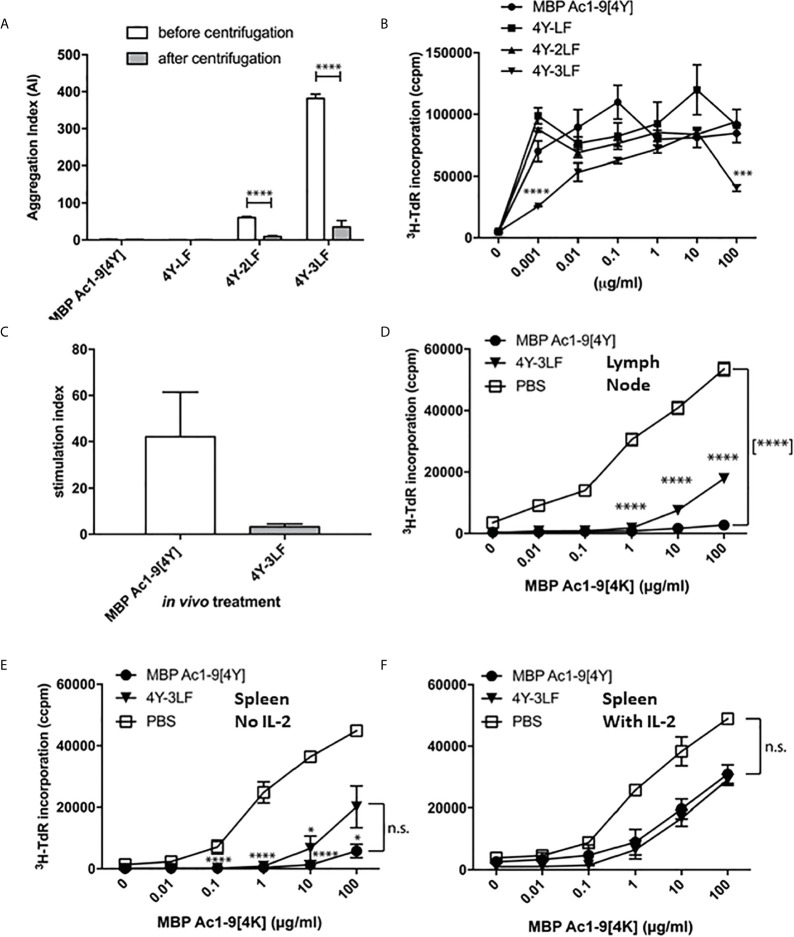
Peptide solubility predicts accessibility to steady state DC and determines tolerance induction: The 4Y peptide was rendered increasingly insoluble by addition of LF motifs. Aggregation index (AI) calculated by the formula AI = 100 x (Abs340/(Abs280-Abs340). All bars show the mean and SEM of eight repeat measurements taken in a single experiment. **(A)** peptides with 2-3 LF motifs were increasingly aggregated but remained antigenic **(B)** as evidenced by tritiated thymidine (^3^H-TdR) incorporation at d3 after addition to Tg4 splenocytes (2x10^5^ per well) *in vitro*. **(C)** shows cell proliferation of Tg4 CD4^+^ T cells simulated *in vitro* with CD11c^+^ cells purified from mice treated with 80µg 4Y or 4Y-3LF SC, as in **Figure 1A**. **(D)** Tg4 mice received escalating doses (see **Figure 3E**) of 4Y (filled circle), 4Y-3LF (filled inverted triangle), or PBS (open square) injections SC every 3-4 days. Inguinal and brachial lymph nodes were isolated three days following the sixth dose and cultured at 5x10^4^ cells per well in a 96–well plate with Ac1–9[4K]. Proliferation was measured by tritiated thymidine (^3^H-TdR) incorporation at 72 hours. Using the same conditions as in **(D)**, **(E)** analyses the response of 5x10^4^ splenocytes per well without IL-2 or following addition of exogenous rhIL-2 at 20 U/ml **(F)**. Graphs show mean of triplicate wells and error bars show SEM (*p ≤ 0.05, **p ≤ 0.01, ***p ≤ 0.001, ****p ≤ 0.0001).

We have investigated the role of peptide solubility by creating a panel of peptides based on the model apitope 4Y, see **Table 1**. These peptides were rendered increasingly hydrophobic by addition of repeated leucine-phenylalanine motifs at the C-terminus. As shown in **Figure 2A**, the more hydrophobic peptides aggregated and lost absorbance following centrifugation. The aggregated peptides still remained antigenic when incubated with Tg4 splenocytes *in vitro* (**Figure 2B**) although the 3LF derivative was significantly weaker at a limiting dose (1 ng/ml).

We have used the CD11c^+^ dendritic cell (DC) targeting approach shown in **Figure 1** to compare targeting by either soluble or relatively insoluble peptides. As shown in **Figure 2C**, the soluble [4Y] peptide was presented by splenic CD11c^+^ dendritic cells (DC) collected 2h after SC administration whereas the relatively insoluble 3LF analogue was barely detectable. This implies that the insoluble peptide is held at the site of injection and prevented from reaching steady state DC in lymphoid organs. We then used the previously described dose escalation protocol (20) to compare the ability of soluble versus insoluble peptides to induce T cell anergy in the Tg4 model. As shown in **Figures 2D, E**, lymph node cells or splenocytes from 4Y treated mice were unable to proliferate *in vitro* when stimulated with the Ac1-9 peptide. The majority of these cells were anergic as evidenced by their ability to proliferate in the presence of exogenous IL-2 (**Figure 2F**). Cells from mice treated with the 3LF analogue were partially suppressed and only marginally more proliferative in the presence of IL-2. These results imply that insoluble peptides are significantly less effective in their ability to induce anergy in CD4 T cells either because the levels of effective peptide reaching APC in lymphoid organs is decreased substantially (**Figure 2C**) or potentially because the peptide is processed at the site of injection before being carried to lymphoid organs by skin APC.

**Figure 3:**
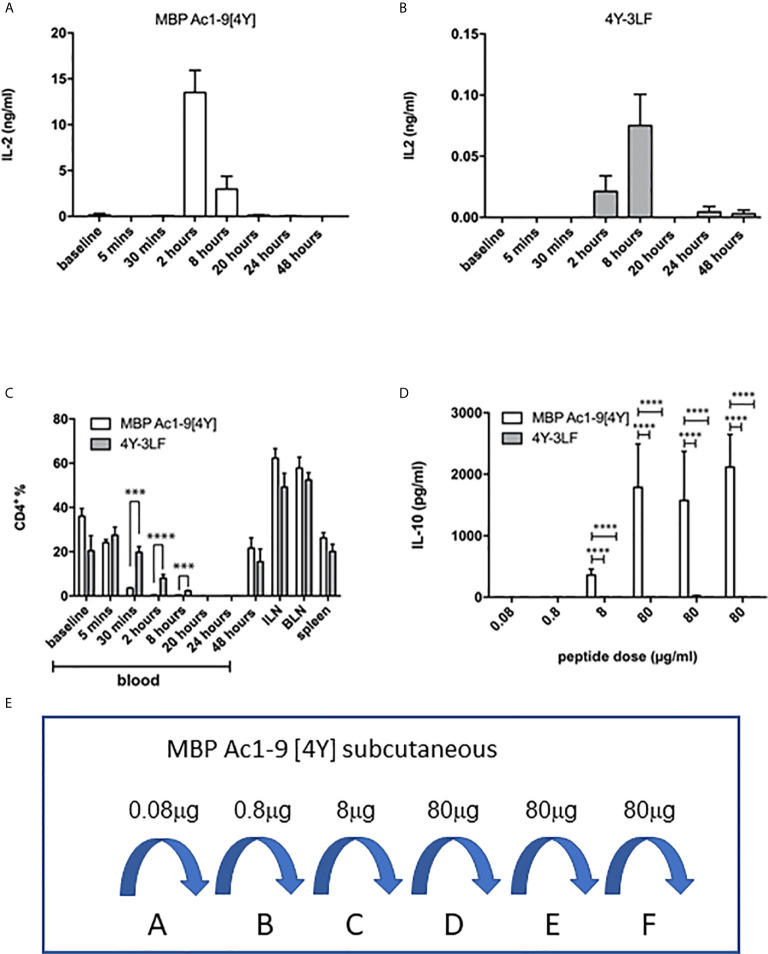
Impact of soluble versus insoluble peptide treatment on T cell function in Tg4 mice. A single SC dose of soluble 4Y (80µg) induces rapid and high levels of IL-2 into the serum of Tg4 mice **(A)** when compared to insoluble 4Y-3LF **(B)**. For the data shown in **(C)**, Tg4 mice received a SC injection of 80µg MBP Ac1-9[4Y] or 4Y-3LF. Blood was removed from the tail vein at the specified times following injection, baseline was taken prior to peptide injection and the cellular fraction was stained for flow cytometry. Inguinal lymph node (ILN), brachial lymph node (BLN) and spleen were collected at 48–hours. Percentages of CD4^+^ cells were gated on live singlets. Dose escalation of 4Y peptide given SC using the protocol shown in (3E) induced high levels of IL-10 secretion into blood **(D)** that was not seen in mice treated with 4Y-3LF (open bars = 4Y; filled bars = 4Y-3LF), error bars show SEM. (*p ≤ 0.05, **p ≤ 0.01, ***p ≤ 0.001, ****p ≤ 0.0001). **(E)** shows the dose escalation protocol used for tolerance induction in **(D)**. Tg4 mice were given SC injections of PBS containing the indicated doses of 4Y peptide given every 3^rd^ or 4^th^ day (2 doses per week).

The efficacy of presentation of soluble versus insoluble peptide was compared *in vivo* by measuring the kinetics of IL-2 secretion, detectable in the blood, in response to a single dose of 4Y versus 3LF. As shown, in **Figure 3A**, SC injection of 4Y led to a peak of IL-2 secretion at 2h, as previously shown for both IN (22) and SC (20) routes. The level of serum IL-2 induced by the poorly soluble 3LF peptide did not peak until 8h after SC injection and was ~200 fold lower than that induced by the soluble peptide (**Figure 3B**). Kearney and colleagues previously noted that antigen-specific T cells disappear from the circulation when activated with soluble peptide (28). This approach showed that the administration of either soluble or insoluble peptide did not impact the % of CD4 cells in lymphoid organs (**Figure 3C**). In response to the 4Y peptide, however, CD4 cells in blood disappeared significantly by 30 mins and were virtually undetectable at 2h. Blood CD4 cells were also reduced in response to the 3LF peptide but this took between 2 and 8h.

The ultimate effect of 4Y administration in the Tg4 model is the induction of IL-10 secreting Tr1 cells (20, 22). For tolerance induction we used the previously described dose escalation protocol whereby the dose of peptide injected every 3^rd^ or 4^th^ day is increased 10-fold before giving repeated maximal doses (20) as shown in **Figure 3E**. **Figure 3D** shows the levels of IL-10 detectable in the serum of Tg4 mice treated with 4Y versus 3LF. No IL-10 was detectable in mice treated with 3LF. This observation was supported by intracellular cytokine staining of spleen cells after the final 80 µg dose of peptide. This showed that >20% of CD4 cells from [4Y] treated mice produced IL-10 whereas cells from mice treated with 3LF were close to background levels.

We have analyzed the role of solubility in apitope-induced tolerance using other model peptides. For example, we have designed tolerogenic pan-DR binding apitopes from the dominant T cell epitopes of thyroid stimulating hormone receptor (TSHR), the target of activating antibodies in Graves’ hyperthyroid disease. Appropriate analogues with optimized antigenicity and solubility have been tested in HLA-DR3 transgenic mice (6) and in humans through a phase 1 clinical trial in patients with mild to moderate Graves’ disease (8). The 5D peptide represents amino acids 81-95 of the extracellular domain of TSHR (**Supplementary Table 1**). Analogue 5D-K1 spans residues 82-95 with 4 lysine residues at the N- and 3 lysines at the C-terminus while 5D-K16 spans residues 82-93 with 3 lysine residues at N- and C-termini. **Supplementary Figures 1A–C** shows solubility analysis of the 5D epitope compared to 5D-K16 apitope as measured by aggregation and dynamic light scattering. We then tested the kinetics of peptide delivery to steady state DC *in vivo* (**Supplementary Figure 1D**). Analysis of repeat experiments (n=8) shows that the 5D-K16 peptide is presented by splenic DC within 5 mins following SC injection as evidenced by IFN-gamma secretion from 5D-specific T lymphocytes. Presentation of this peptide reached a peak at 15 mins after SC injection and remained readily detectable for up to 1h.

### Impact of Soluble Peptide Therapy on T Cell Signaling and Function

**Figure 4:**
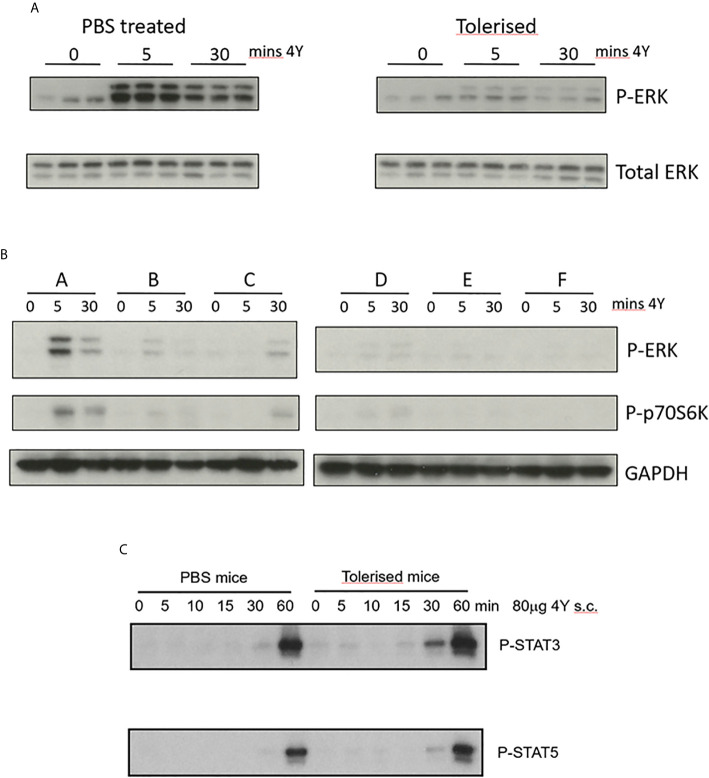
Impact of peptide induced tolerance induction on cell signaling **(A)** CD4^+^ T cells were enriched from spleens of naïve (PBS treated) versus tolerized Tg4 mice at the indicated times following challenge with 80µg MBP Ac1-9[4Y] SC, cell extracts prepared and Western blots stained for either phospho-ERK or total ERK protein. **(B)** CD4^+^ T cells collected at the indicated time after Tg4 mice were tolerized by dose escalation as shown in **Figure 3E**. Western blots were stained for phosphor-ERK and phosphor-p70S6K using GAPDH as loading control. **(C)** CD4^+^ T cells were enriched from spleens of naïve versus tolerized Tg4 mice at the indicated times following challenge with 80µg MBP Ac1-9[4Y] SC, cell extracts prepared and Western blots stained for either phospho-STAT3 or phospho-STAT5.

We have shown previously that the optimal protocol for tolerance induction in the Tg4 mouse involves dose escalation of the 4Y model apitope from 0.08 to 80µg with peptide being injected SC every 3^rd^ or 4^th^ day (**Figure 3E**) (20). Here we compare ERK phosphorylation in naïve versus tolerized Tg4 mice. Mice were treated with an escalating dose of 4Y in PBS (**Figure 3E**) or with PBS alone. Splenic CD4 cells from PBS treated mice responded to a single dose of 80µg 4Y with phospho-ERK appearing rapidly following SC injection (**Figure 4A**) and declining within 30 mins. ERK phosphorylation was substantially suppressed in the spleens of mice rendered tolerant by SC injection of 4Y. Furthermore, as shown in **Figure 4B**, activation of both phospho-ERK and phospho-p70S6K signaling pathways was effectively switched off following two doses of peptide since the level of phosphorylation of both ERK and p70S6K was diminished substantially. Attenuation of ERK and p70S6K pathways did not alter responsiveness of Tg4 T cells to cytokine signaling as shown by sustained activation of both STAT3 and STAT5 pathways *in vivo* **Figure 4C**.

### Peptide Induced Tr1 Cells Migrate Into Tissues and Control Inflammation

**Figure 5:**
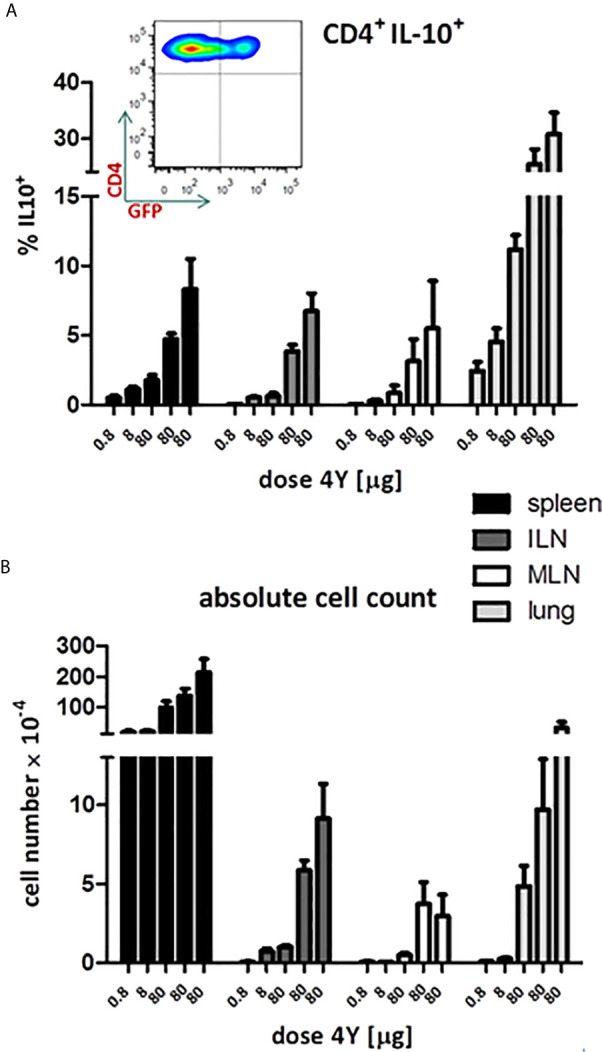
IL-10 production in tiger-Tg4 mice during dose escalation immunotherapy. Tg4GFP/IL10 mice were treated with 4Y according to the dose escalation protocol in **Figure 3E**. Different organs were isolated from 6 mice over 2 independent experiments (ILN=inguinal lymph nodes; MLN=mediastinal LN). The inset shows a representative cytometry plot from a spleen after the last dose of 4Y gated on live single CD4+ T cells. Bar graphs show mean ± SEM. **(A)** represents % of GFP^+^ cells among gated CD4^+^ cells whereas **(B)** shows the absolute number of GFP^+^ IL-10 producing CD4^+^ cells.

Our previous analysis of the IL-10^+^ CD4^+^ T cells induced by SC administration of the model apitope 4Y (20) showed that these cells have the same phenotype, expressing CTLA-4, Tim3, TIGIT and Lag3, as Tr1 cells generated *in vitro* (29). In order to monitor the appearance and migration of IL-10 secreting cells following administration of soluble peptide, we have used the tiger-IL-10 reporter mouse (18) backcrossed onto the Tg4 mouse background, as described previously (20). The tiger mouse, developed by Flavell and colleagues, is a green fluorescent protein (GFP) knock-in mouse and a valuable tool for IL-10 analysis whereby multiple rounds of TCR stimulation generates cells highly committed to produce IL-10 *in vivo* (18). The tiger mouse has been used previously to study Tr1 cell differentiation (16, 20, 30) with IL-10 expressing cells upregulating expression of inhibitory receptors such as Lag3. Here we reveal the gradual increase in proportion of IL-10^+^ cells in tiger-Tg4 mice following treatment with escalating doses of 4Y peptide (see **Figure 3E**). The proportion of IL-10 producing cells increased to between 5 and 10% in spleen and lymph nodes but reached substantially higher levels in lungs of treated mice (**Figure 5A**). Absolute numbers of IL-10 producing cells were higher in spleen and lung when compared with lymph nodes (**Figure 5B**).

**Figure 6:**
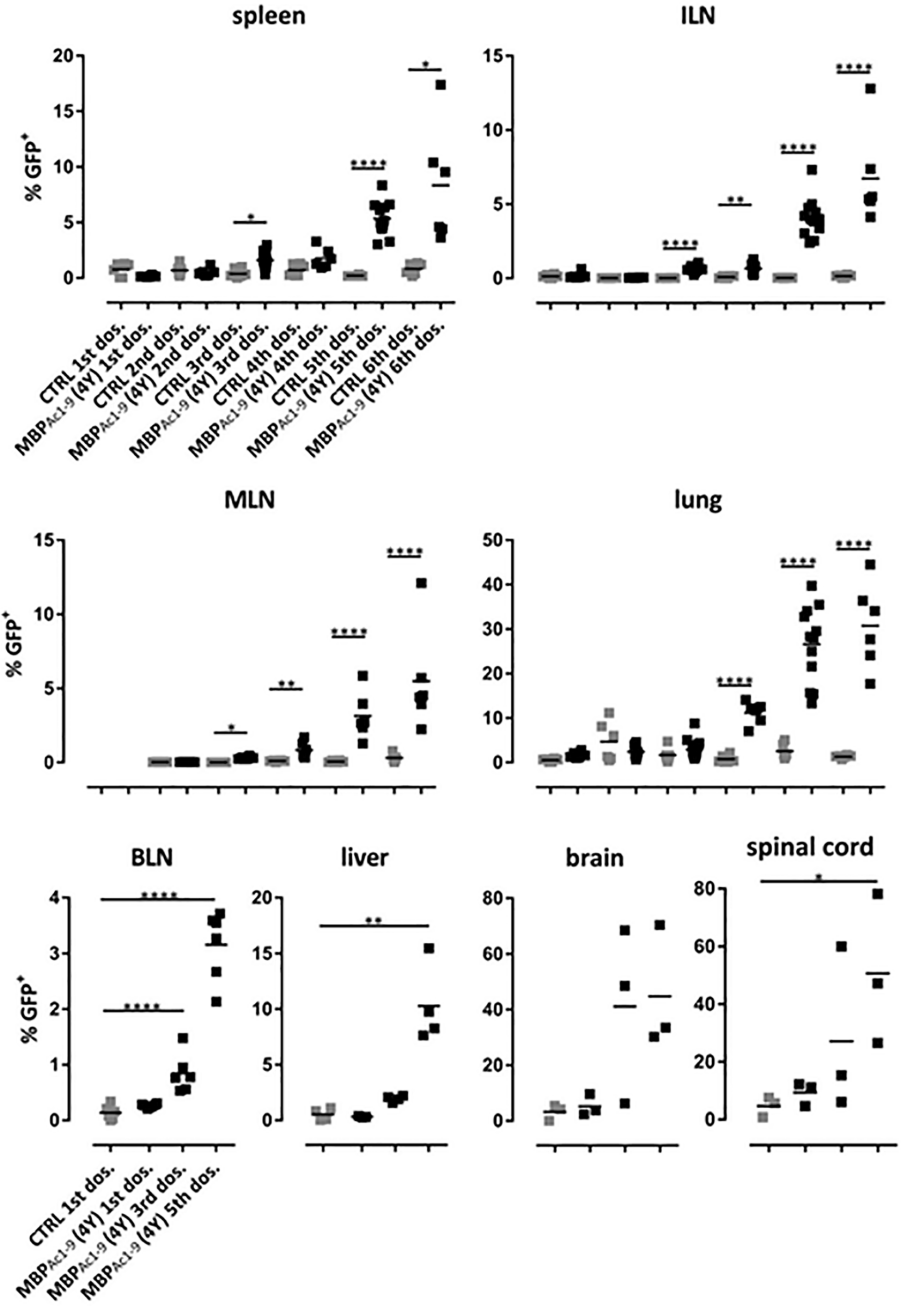
Distribution of IL-10 producing CD4^+^ T cells during the course of tolerization. tiger-Tg4 mice were treated with 4Y peptide by dose escalation as shown in **Figure 3E**. Two hours after the indicated dose animals were perfused with PBS and organs were isolated. After gating on viable, single CD4^+^ T cells, IL-10^+^ T cells were investigated by flow cytometry. Horizontal lines indicate the mean (*p ≤ 0.05, **p ≤ 0.01, ***p ≤ 0.001, ****p ≤ 0.0001). In the spleen, ILN (inguinal lymph nodes), MLN (mediastinal LN), lung and BLN (brachial LN) each dot represents one individual. In the liver, in some cases cells from two animals were combined due to low cell numbers while in the CNS compartment each dot represents two individuals.

**Figure 6** shows the distribution of IL-10 secreting cells in different tissues. Levels of these cells increased with increasingly higher doses of peptide or, in other words, the proportion of IL-10 secreting cells increased with higher signal strength. Importantly, administration of soluble peptide led not only to increasingly higher proportions of Tr1 cells in spleen and lymph nodes (20) but also to high proportions of IL-10^+^ CD4^+^ cells in brain and spinal cord. These results show that SC administration of peptides leads to migration of IL-10^+^ regulatory cells, the majority of which were Tr1 cells to extra-lymphoid tissues including liver and the CNS. As shown previously (20), the IL-10^+^ cells induced by 4Y expressed PD-1, Tim3 and TIGIT. The majority of IL-10^+^ cells were Foxp3^-^ although there was also an increase in the % of Foxp3^+^ cells in lymphoid organs (20). Both the Foxp3^+^ and majority Foxp3^-^ (Tr1) cell populations entering the CNS displayed upregulated IL-10, PD-1, Tim3 and TIGIT (data not shown).

**Figure 7:**
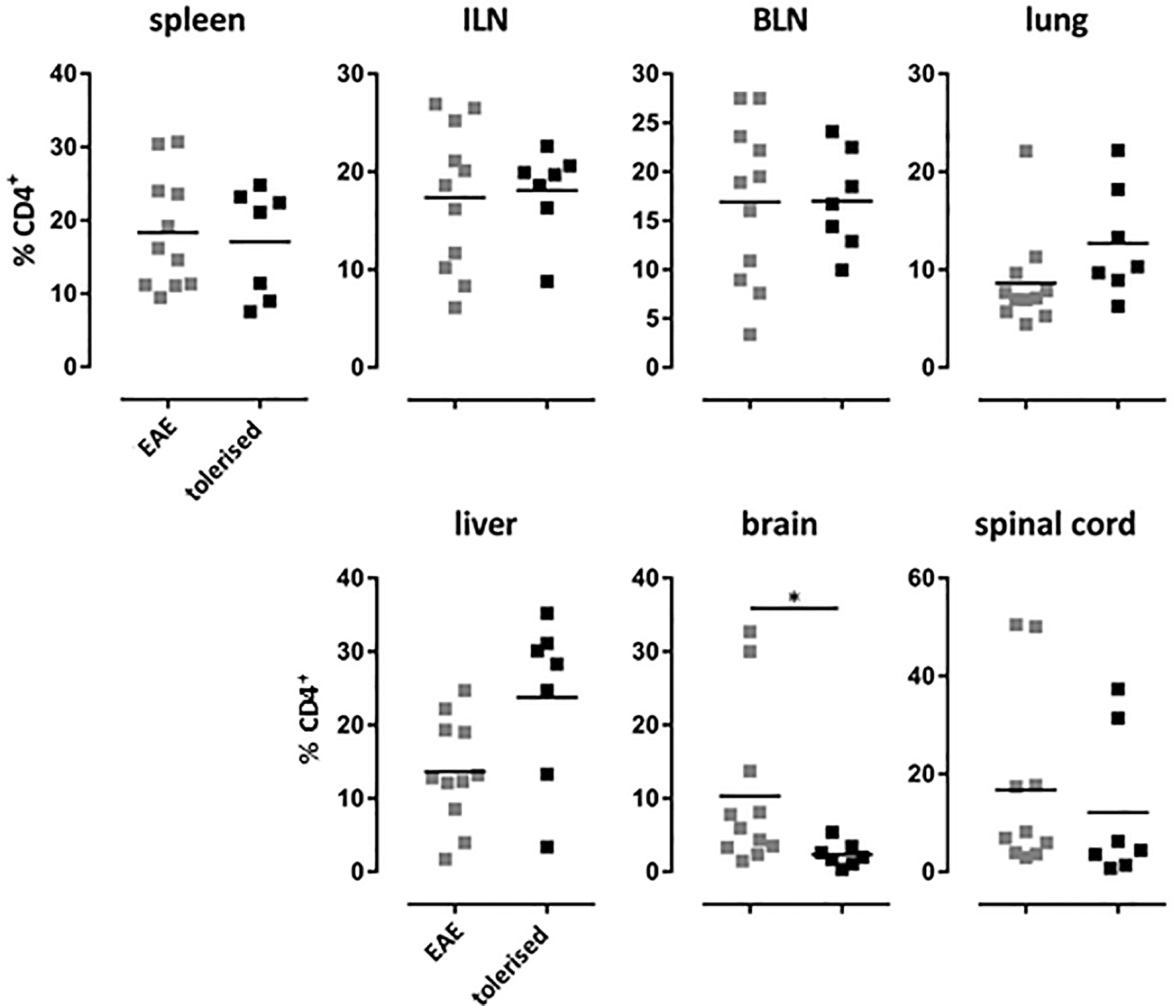
Distribution of CD4^+^ T cells in EAE after tolerization. Tg4 mice were treated with PBS or 4Y by dose escalation (**Figure 5A**) before the induction of EAE with their cognate peptide in CFA, and pertussis toxin given on days 0 and 2. Animals were analyzed at the peak of disease when mice treated with PBS showed complete hind limb paralysis (grade 3) and EDI-treated animals showed no sign of disease. Gated on single cells. Horizontal lines indicate mean (*p ≤ 0.05, **p ≤ 0.01, ***p ≤ 0.001, ****p ≤ 0.0001). Each dot represents one individual.

We have shown previously that treatment of Tg4 mice with soluble 4Y peptide led to protection from experimental autoimmune encephalomyelitis (EAE) that was both dose (20) and IL-10 dependent (31). Furthermore, we have shown that 4Y treatment after disease induction blocks disease progression in the Tg4 model (20). Here, EAE was induced in mice that had previously received 4Y by dose escalation or PBS as control. Tissues were harvested from mice with grade 3 EAE or simultaneously from mice protected by therapy with SC 4Y peptide. As shown in **Figure 7**, there was no significant difference between the proportion of CD4 cells in the lymphoid organs of control mice with EAE versus tolerized mice with no signs of disease. There was, however, a significant reduction in the % of cells found in the brain tissue of protected mice. Furthermore, analysis of cells recovered from the CNS of tiger-Tg4 mice, in an EAE experiment, were consistent with the results shown in **Figure 6**. There was no difference between 4Y-tolerized versus control mice in the % of GFP^+^ cells in lymphoid tissues, lung and liver at the peak of EAE. However, 9% of the few cells in the brain of tolerized, disease-free mice were IL-10^+^ whereas only 3% of cells were GFP^+^ in control mice with EAE. This implies that peptide treatment reduces the migration, persistence and/or proliferation of potentially pathogenic cells in the CNS while allowing passage of immunoregulatory IL-10-secreting cells into the tissue.

## Discussion

Our previous studies have shown that soluble peptides based on CD4^+^ T cell epitopes can protect from autoimmune and allergic diseases (3). This results in the generation of both linked and bystander suppression (32), suppression of inflammatory cytokine production and induction of IL-10 (22, 31). Importantly, apitope treatment has been shown to both protect from and suppress ongoing disease in the EAE model of multiple sclerosis (20, 33, 34). The work described here addresses key mechanisms in the process of tolerance induction with the model apitope 4Y and the function of the resulting Tr1 cells by focusing on the nature of the APC, peptide solubility, the impact on cell signaling and migration of the induced IL-10 secreting, predominantly Tr1 cells.

In theory, soluble CD4^+^ T cell epitopes administered SC could bind to many APC ranging from skin resident DC, to lymphoid DC, B cells and monocytes. It is important to appreciate that small, soluble peptides will have a short half-life *in vivo*. Such peptides will enter the blood and lymph and will be excreted rapidly. Nevertheless, we have shown that the model apitope 4Y is found on splenic APC within minutes of injection and is readily detectable beyond 2h hours later (23). The question is which APC do soluble peptides bind to and which APC are responsible for tolerance induction? Here we show that soluble peptides preferentially bind steady state CD11c^+^ DC but not B cells or monocytes and that these cells induce anergy on repeated administration *in vivo*. Why should soluble peptides selectively target steady state DC? Stern and colleagues previously showed that immature, steady state DC from both mouse and human have peptide-receptive MHC at the cell surface (35, 36). This exceptional property of steady state DC can be accounted for by the lower level of endosomal acidification in immature, steady state DC which prevents efficient MHC loading (37).

We propose that peptides need to be soluble to induce optimal tolerance through induction of anergy and promotion of IL-10. Furthermore, we show here that soluble peptides target steady state DC in spleen and lymph nodes whereas insoluble analogues of the same peptides fail to do so. Two observations suggest that peptides target steady state DC in lymphoid organs rather than binding skin DC that then migrate to draining lymph nodes. First, we show that peptide can be detected on steady state DC within 5 mins following SC injection and secondly, that this leads to immediate TCR triggering as evidenced by ERK phosphorylation. The rapid appearance of peptide on steady state DC is inconsistent with migration of DC from skin which in mice is known to occur between 6 and 24h following skin sensitization (38). SC injection of poorly soluble peptide led to a markedly delayed and a substantially lower level of T cell activation *in vivo* (<1%). Data on IL-2 production and CD4 cell levels in blood is, therefore, consistent with migration of peptide carrying DC from skin or alternatively the slow release of low levels of peptides from insoluble complexes. Either way, the level of peptide delivered is low as evidenced by the low levels of IL-2 induced and this is insufficient to promote IL-10 production *in vivo*.

As shown previously, the first encounter with soluble peptide leads to transient T cell activation but this is followed by cell anergy and the generation of a Tr1 phenotype (22, 39). Here we show that peptide induced tolerance correlates with suppression of ERK phosphorylation while cytokine receptor signaling is unaffected, reflecting a selective TCR proximal block in signaling. It is then reasonable to ask how this block in TCR signaling promotes differentiation of IL-10 secreting Tr1 cells. Recently, we have shown that the peptide induced differentiation of Tr1 cells such as that induced by the model apitope 4Y arises from the combined impact of a membrane proximal block in signaling combined with epigenetic priming of a set of tolerance associated genes (11). Bevington and colleagues provide evidence that Tr1 cell differentiation, arising from peptide immunotherapy, involves a combination of epigenetic priming among a set of tolerance associated genes along with a membrane proximal block in signaling that deprives inflammatory genes of the transcription factors they require for transcription. Genes associated with inflammation depend on transcription factors arising from strong signaling in naïve and effector T cells. These inflammation-associated genes are not epigenetically primed and are not expressed in tolerant cells because they require an elevated threshold of signaling for their induction. Conversely, epigenetic priming creates an accessible chromatin environment at immune-suppressive genes including Ctla4 and Il10 such that they can be induced in tolerant Tr1 cells in the presence of reduced levels of TCR/CD28 signaling. Together with the data in this paper, this novel model of T cell anergy explains how soluble peptide administration results in tolerance and the generation of regulatory cells with a Tr1 phenotype.

Although we have shown that the model apitope 4Y induces regulatory Tr1 cells, we did not know whether these cells and other IL-10 producing regulatory populations would migrate from lymphoid tissues to control autoimmunity in other organs. Here we show that up to 10% of CD4 cells recovered from lymphoid organs of Tg4 mice treated with tolerogenic peptide express IL-10. A higher % of the remarkably high number of cells recovered from the lung expressed IL-10 and an even higher % of cells migrating into CNS tissue were IL-10 positive at later stages of tolerance induction. It could be argued that this represents a progression from tolerance induction in lymphoid organs through migration of Tr1 cells to peripheral tissues and accumulation in CNS tissues. Our evidence suggests, however, a special role for lung tissue since levels of IL-10+ve cells were similar in liver and lymphoid tissue but much higher in lung. This is reminiscent of previous studies by Odoardi and colleagues who showed that activated T cells specific for myelin basic protein (MBP) migrate to lung tissue prior to entering the CNS (40). They suggested that T cells become licensed in the lung to enter the CNS. The implication of our findings is that such licensing could also impact the migration and accumulation of MBP specific Tr1 cells.

In conclusion, here we have addressed some of the key remaining questions relating to the use of apitopes based on T cell epitopes for antigen-specific immunotherapy and for the resulting Tr1 cell induction. The importance of peptide solubility relates to the need for tolerogenic peptides to flow rapidly to lymphoid organs and bind steady state DC. Peptide bearing DC reproduce the anergy inducing properties of injected peptides by cell transfer. We confirm that the tolerogenic effect of soluble peptides results in a block in cell signaling that selectively suppresses TCR-mediated signaling while leaving cytokine signaling intact. This complements recent cell signaling studies in the Tg4 model that revealed a novel mechanism whereby selective epigenetic priming of tolerance-associated genes permits their transcription despite the suppression of TCR-mediated signaling (11). Finally, we use the tiger-Tg4 IL-10 reporter mouse to demonstrate that the Tr1 cells induced by tolerogenic peptides migrate from lymphoid organs and can traffic to the CNS. Our results provide a mechanistic basis for the use of tolerogenic peptides for treatment of human autoimmune conditions. Recent clinical trials of immunotherapy with apitopes in Graves’ disease and MS show that tolerogenic peptides are well tolerated with promising evidence of efficacy (7, 8).

## Data Availability Statement

The original contributions presented in the study are included in the article/**Supplementary Material**. Further inquiries can be directed to the corresponding author.

## Ethics Statement

The animal study was reviewed and approved by Animal experiments in the UK were carried out under a UK Home Office Project License (30-3195) and approved by the University of Bristol ethical review committee. Mice were bred and kept under specific pathogen‐free conditions. HLA-DR3 mice studies were approved by the ‘Ethical Committee for Animal experiments’ (ECD) at Hasselt University, Belgium.

## Author Contributions

ERS, AW, EVH, BRB, STHN and HBS helped design peptides and conduct experiments shown in **Figures 1–7**. ERS, SA, ES, BH and LJ designed the peptides and helped conduct the experiments shown in **Supplementary Table 1** and **Supplementary Figure 1**. DCW provided grants, designed experiments and wrote the paper. All authors contributed to data interpretation and editing the paper.

## Funding

This work was supported by the University of Birmingham; a Wellcome Trust Programme Grant (091074/Z/09/Z); MRC-Case Studentship (MR/K015990/1) and Marie Curie Fellowship (ITN NeuroKine; 316722) from the European Union in partnership with Apitope International NV and by grants to Apitope from Agentschap voor lnnovatie door Wetenschap en Technologie (IWT 120512), Belgium and an EU Seventh framework (FP7) program, agreement 602779 DAVIAD.

## Conflict of Interest

DW is Professor of Immunology at the University of Birmingham and CSO and Founder of Apitope International NV; ES, BH and LJ are or were recent employees of Apitope International NV.

The remaining authors declare that the research was conducted in the absence of any commercial or financial relationships that could be construed as a potential conflict of interest.

## Supplementary Material

Supplementary Figure 1 Optimization of solubility in a dominant TSHR epitope. Centrifugation of the 5D peptide of TSHR reveals that this peptide is of poor solubility when compared with the modified 5D-K16 epitope **(A)** as evidenced by removal of aggregates **(B)**. Dynamic light scattering shows the presence of heterologous aggregates in 5D when compared with the uniform nature of the soluble 5D-K16 analogue **(C)**. 5D-specific T cells from draining lymph nodes and spleen of DR3 mice immunized with peptide 5D in CFA responded to CD11c^+^ DC from HLA-DR3 transgenic mice by secreting interferon gamma as early as 5 min following SC injection of the 5D-K16 analogue **(D)**. **Supplementary Figure 1D** shows the results of from 8 individual CD11c^+^ DC enrichment experiments, error bars show SEM (* = p ≤ 0.05, ** = p ≤ 0.01, *** = p ≤ 0.001, **** = p ≤ 0.0001).
