# Supplementary figures and images for: The Mechanism of Action of Antigen Processing Independent T Cell Epitopes Designed for Immunotherapy of Autoimmune Diseases
Source: Front Immunol. 2021 Apr 14;12:654201. doi: 10.3389/fimmu.2021.654201 (PMC8079784; doi:10.3389/fimmu.2021.654201)

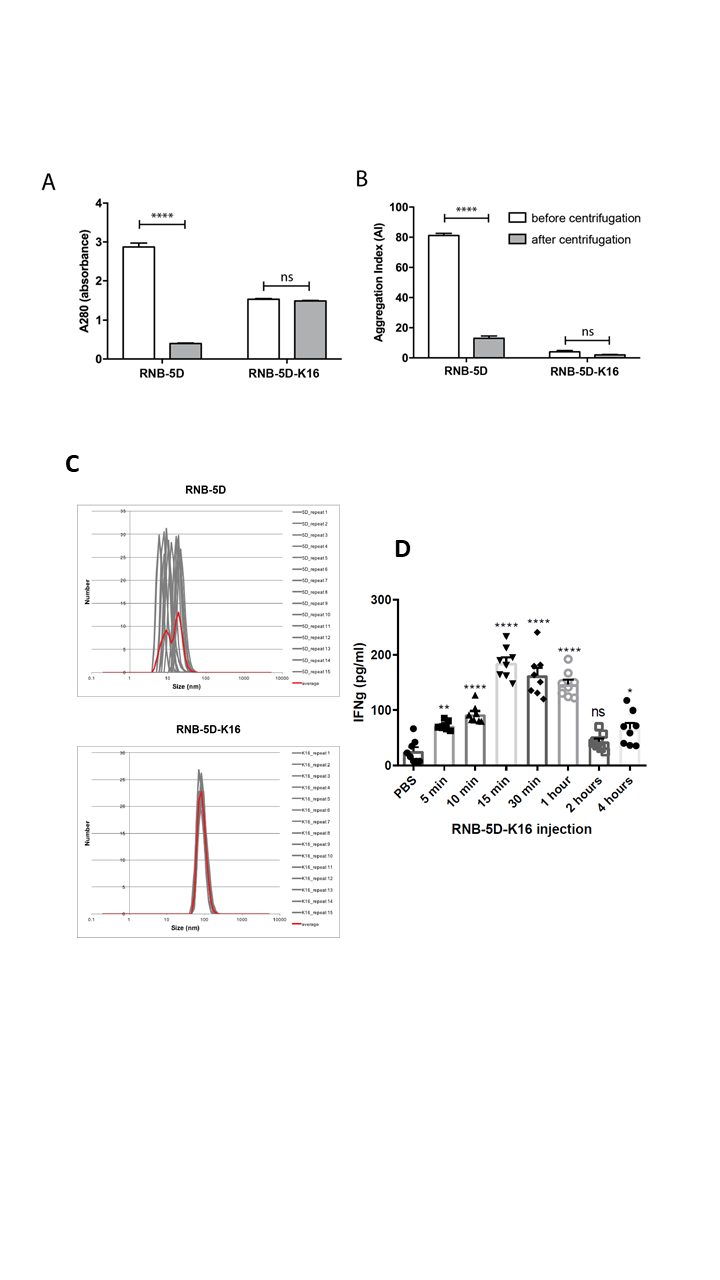

Supplement: Supplementary Figure 1 — Optimization of solubility in a dominant TSHR epitope. Centrifugation of the 5D peptide of TSHR reveals that this peptide is of poor solubility when compared with the modified 5D-K16 epitope (A) as evidenced by removal of aggregates (B). Dynamic light scattering shows the presence of heterologous aggregates in 5D when compared with the uniform nature of the soluble 5D-K16 analogue (C). 5D-specific T cells from draining lymph nodes and spleen of DR3 mice immunized with peptide 5D in CFA responded to CD11c+ DC from HLA-DR3 transgenic mice by secreting interferon gamma as early as 5 min following SC injection of the 5D-K16 analogue (D). Supplementary Figure 1D shows the results of from 8 individual CD11c+ DC enrichment experiments, error bars show SEM (* = p ≤ 0.05, ** = p ≤ 0.01, *** = p ≤ 0.001, **** = p ≤ 0.0001). [file Image_1.tif]
