# Supplementary material for: The Mechanism of Action of Antigen Processing Independent T Cell Epitopes Designed for Immunotherapy of Autoimmune Diseases
Source: Front Immunol. 2021 Apr 14;12:654201. doi: 10.3389/fimmu.2021.654201 (PMC8079784; doi:10.3389/fimmu.2021.654201)
Supplement: Supplementary file 2 [file Table_1.docx]

Supplementary Table 1

|  | Peptide name | Sequence | GRAVY score |
| --- | --- | --- | --- |
| Native TSHR sequence 81-95 | 5D | IYVSIDVTLQQLESH | 0.28 |
| Minimal 5D epitope (82-93) optimised for solubility | 5D-K16 | KKKYVSIDVTLQQLEKKK | -1.09 |
| 5D (82-95) optimised for solubility | 5D-K1 | KKKKYVSIDVTLQQLESHKKK | -1.31 |

Supplementary Table 1: Properties and sequences of modified TSHR peptides
